# Comparative Evaluation of the Effectiveness of 40% Miswak Mouthwash and 0.12% Chlorhexidine Mouthwash in Treating Gingivitis: A Blinded, Randomised Clinical Trial

**DOI:** 10.3290/j.ohpd.b1179501

**Published:** 2021-04-09

**Authors:** Sumayah Al-Mahmood, Dunia Wadeea Sabea

**Affiliations:** a Lecturer, Basic Sciences Department, College of Dentistry, Al-Iraqia University, Baghdad, Iraq. Study concept and design, data collection and analysis, wrote, reviewed and finally approved the manuscript, prepared the submission.; b Assistant Professor, Oral Pathology Department, College of Dentistry, Al-Iraqia University, Baghdad, Iraq. Wrote, reviewed and finally approved the manuscript, prepared the submission.

**Keywords:** chlorhexidine, gingivitis, miswak, mouthwash, oral health

## Abstract

**Purpose:**

To assess the effectiveness of a 40% miswak compared to a 0.12% chlorhexidine mouthwash.

**Materials and Methods:**

A total of 60 patients aged 20-55 years who attended the Periodontics Clinics at the College of Dentistry, Al-Iraqia University, Baghdad, Iraq, were allocated into 2 groups to use either 40% miswak mouthwash or 0.12% chlorhexidine gluconate Kin Gingival (Laboratorios KIN) twice daily for 2 months. Gingival, bleeding, and plaque indices were assessed.

**Results:**

There were statistically significant differences between the effectiveness of miswak and chlorhexidine mouthwashes in terms of gingivitis. The means of gingival, bleeding, and plaque indices using miswak mouthwash were 1.2, 0.4, and 0.53, respectively, i.e. indicating lower effectiveness, than when 0.12% chlorhexidine mouthwash was used (0.87, 0.43, 0.23, respectively).

**Conclusion:**

Miswak mouthwash is a good oral hygiene agent especially for long-term use even if its efficacy is lower than chlorhexidine mouthwash.

Gingivitis is the mildest form of gum disease and is elicited by the accumulation of plaque on the tooth and the soft tissue adjacent to the tooth. Accumulation of dental biofilm has been established as the initial cause of this disease.^19^ It is characterised by redness of the gum margins, edema, bleeding upon brushing, and loss of periodontal attachment.^19^ Gingivitis exists in both acute and chronic forms.^19^ Acute gingivitis is can be caused by trauma, micro-organisms, and specific infections, while chronic inflammation is related to a bacterial biofilm which covers the gingiva and the adjoining teeth.^19,20,26^ Elimination and control of plaque are very important in the maintenance of periodontal health.^27^ Mechanical and chemical plaque control, such as toothbrushing, daily flossing, and regular oral hygiene, can help in the control and prevention of dental plaque.^27^

Mouthwashes or dentifrices containing chemical or herbal substances are adjunctive means of improving oral hygiene by preventing bacterial adhesion to dental surfaces or by inhibiting the bacterial growth rate in plaque.^8,24^ Chlorhexidine (CHX) mouthwash is the most effective antimicrobial and antiplaque agent. However, its use is limited due to side effects such as tooth staining and staining of certain restorations with prolonged usage, ulceration/erosions, oral mucosa irritation, a sensation of tongue burning, hypersensitivity, stomatitis and taste alterations.^7,25^ Chlorhexidine mouthwash has a tremendous effect when it is used as a mouthwash for long periods; therefore, the Food and Drug Administration (FDA) recommend limiting the use of chlorhexidine mouthwash to no longer than 6 months to minimise its adverse effects. Additionally, instead of chemical mouthwashes, the World Health Organization (WHO) has encouraged the use of herbal extracts and natural plant mouthwashes, such as miswak.^16^ Miswak mouthwash is a herbal mouthwash that is available locally and culturally accepted. It is prepared from a tree called *Salvadora persica* (Arak) which grows in Africa and west India. Because of the potent properties of miswak, it has been used in different forms, e.g. toothpaste, extract and sticks. In developing countries, it is widely used as a traditional practice because of its availability and low cost.^10^

In this study, we compared the effects of two mouthwashes – one with miswak and the other with chlorhexidine – in treating gingivitis, hypothesising that miswak would yield better results than CHX.

## Materials and Methods

### Study Design and Setting

Sixty patients were enrolled in this study, 36 males and 24 females who attended the Periodontics Clinics at the College of Dentistry, Al-Iraqia University from 15th January to 15th March 2020. All of them were clinically examined, their medical history was taken, scaling and polishing were performed, oral hygiene instructions were given, and then they were randomly assigned to one of the following mouthwash groups: miswak mouthwash and 0.12% chlorhexidine gluconate mouthwash for seven days.^9^ The study period was determined according to a study done by Al-Sultan^9^, and was approved by the Scientific Committee of the College of Dentistry, Al-Iraqia University, Baghdad, Iraq.

### Ethical Approval

All authors declared that all experiments were examined and approved by the appropriate ethics committee from the College of Dentistry, Al-Iraqia University and were thus performed in accordance with the ethical standards laid down in the 1964 Declaration of Helsinki. This trial was retrospectively registered in ClinicalTrials.gov (ID number: NCT04607785).

### Data Measurement

The baseline data were measured after scaling and polishing. Mechanical plaque was not controlled during the study; brushing at least once/day was recommended.

### Randomisation Process

#### Participants

**Fig 1:**
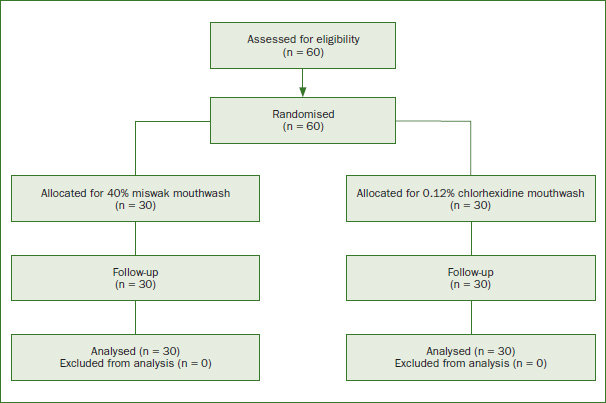
CONSORT study design.

This study included 60 patients with gingivitis (ages: 20–55 years). Figure 1 shows the number of eligible patients, randomisation, allocation, follow-up, and analysis according to CONSORT.

The inclusion criteria were: adults with at least 20 natural teeth in the mouth; existence of lateral maxillary teeth; existence of central teeth; existence of mandibular teeth. The exclusion criteria were: patients with fixed or removable oral appliances, history of drug use, patients had no systemic disease, pocket depth <3 mm, smokers, pregnancy, history of allergy.

The study was registered with ClinicalTrials.gov in October 2020. However, the study started on January 15, 2020, the primary completion was on March 15, 2020, and the study was completed on March 15, 2020.

### Design

- Primary purpose: supportive care
- Study phase: early phase 1
- Interventional study model: parallel assignment
- Number of arms: 2
- Masking: single (participant)
- Enrollment: 60
- Sponsor: Al-Iraqia University

### Arms and Interventions

#### Miswak mouthwash group

Miswak sticks were bought from local markets in Baghdad, washed with cold water, dried, and then crushed into powder. Later, 7 g of the miswak powder were added to 350 ml of distilled water in a conical flask and left for 24 h. The solution was then filtered and stored in tightly closed bottles in a cool place.^11^ The dosage was twice daily for two months.

#### Chlorhexidine mouthwash group

A 0.12% chlorhexidine gluconate mouthwash (chlorhexidine gluconate Kin Gingival; Kin Laboratories; Barcelona, Spain) was used 2x/day for 2 months.

### Outcome Measures

#### Primary Outcome Measures

- Inflammation of gingiva: change in gingival index (time frame: 2 months)
- Bleeding from gingiva: change in index (time frame: 2 months)
- Plaque in the gingiva: change in index (time frame: 2 months)
- Gingival index, bleeding on probing index (BOP) and plaque index estimation

Data were collected for the gingival and plaque indices as well as BOP, according to Löe and Silness.^17,18^

### Clinical Examination

First, the gingival colour, degree of inflammation, pocket depths and the amount of bleeding upon probing were checked. Gingival and plaque indices were recorded according to Löe and Silness.^17,18^

The gingival index was scored as follows: 0: absence of gingivitis (normal gingival); 1: mild inflammation, slight change in colour, slight edema; 2: moderate inflammation, redness, edema and glazing; 3: severe inflammation, marked redness and edema, ulceration.

For the plaque index, plaque accumulation was estimated and given a score as for the gingival index: 0: no plaque in the gingival areal; 1: a film of plaque adhering to the gingival margin and the adjacent area of the tooth, the plaque may only be recognised by running a probe a cross the tooth surface; 2: a moderate accumulation of soft deposits within the gingival pocket or on the tooth and gingival margin, this can be seen with the naked eye; 3: an abundance of soft matter within the gingival pocket or on the tooth and the gingival margins.

Gingival and plaque indices were scored from the buccal and lingual sides of the teeth (6/2/4). Bleeding on probing was determined from the bucco-distal to the bucco-mesio aspect of each tooth using a probe.

### Statistical Analysis

Data were expressed as mean±SD and analysed using SPSS v 20 (IBM Analytics; Armonk, NY, USA). ANOVA and the paired t-test were applied to compare the treatment groups. Statistical significance was set at p < 0.05.

## Results

The demographic data (sex and age) of the patients involved in the study are presented in Table 1. Patients who used miswak mouthwash showed a statistically significant reduction in gingival index, bleeding on probing, and plaque indices. We revealed a statistically highly significant difference in mean gingival index mean between miswak and CHX mouthwashes, which decreased from 2.2 to 1.2 (p = 0.0001), and chlorhexidine mouthwash, which decreased from 2.13 to 0.87 (p = 0.0001) for the same index. Furthermore, we found a statistically highly significant difference in mean BOP between miswak and CHX mouthwashes: with miswak, it decreased from 1.5 to 0.4 (p=0.0001); with CHX mouthwash, it decreased from 1.47 to 0.43 (p=0.0001). Moreover, there was a statistically highly significant difference in plaque index mean between miswak and CHX mouthwashes: with miswak, it decreased from 1.57 to 0.53 (p=0.0001); with CHX mouthwash, it decreased from 1.2 to 0.23 (p=0.0001) (Table 2).

**Table 1:** Demographic data of the patients included in this study

Variables		Number	%
Gender	Male	36	60
	Female	24	40
Age (years)	20–30	33	55
	31–40	19	32
	41–50	5	8
	51–60	3	5

**Table 2:** Gingival, BOP, and plaque indices (means ± SD) pre- and post-mouthwash treatment

	Miswak mouthwash (n=30)		CHX mouthwash (n=30)		
Index	Before	After	Before	After	p-value (ANOVA)
Gingival	2.2±0.664	1.2±0.664	2.13±0.681	0.87±0.629	0.225
p-value	0.0001		0.0001		
BOP	1.5±0.688	0.4±0.498	1.47±0.777	0.43±0.504	
p-value	0.0001		0.0001		
Plaque	1.57±0.728	0.53±0.507	1.2±0.407	0.23±0.430	
p-value	0.0001		0.0001		

BOP: bleeding on probing.

Moreover, the miswak mouthwash showed lower efficacy (mean: 1.2, 0.4, 0.53 for gingival index, BOP and plaque index, respectively) than the CHX mouthwash (mean: 0.87, 0.43, 0.23 for gingival index, BOP and plaque index, respectively) (p=0.225). No adverse effects were observed during the study period (Table 2).

## Discussion

Gingivitis and plaque have long been considered to be the predominant etiological factor for developing caries and periodontal diseases.^22^ Adequate dental hygiene is vital to prevent the occurrence of these diseases. This dental hygiene can be achieved by chemical or mechanical means, or by a combination of both. Mouthwash as a plaque control technique should be used along with mechanical methods, such as toothbrushing.^22^ It is necessary to recommend that patients also use dental floss.

In this study, miswak mouthwash showed significantly decreased gingivitis and dental plaque within seven days. Miswak contains many therapeutic components, e.g. silica, which acts as an abrasive material to eliminate stains and plaque; alkaloids, which have a bactericidal effect; tannic acid (tannins), which acts astringently on the oral mucous membrane, thus leading to clinical diminishment of gingivitis; essential oils (volatile oils), which have specific aromatic compounds as well as a carminative and antiseptic effect.^5,10^ Miswak also contains other beneficial substances, e.g.: sulfur compounds, which act bactericidally; baking soda (sodium bicarbonate, NaHCO_3_), which has a mild germicidal action; vitamin C, which helps repair and heal tissues, in addition to having minor abrasive characteristics; fluoride, which protects the enamel surface of susceptible sites, such as fissures, pits, and interproximal areas from caries; and elevated concentrations of chloride that inhibit calculus formation.^5^ In addition, the miswak mouthwash showed antimicrobial activity against different types of bacteria: *Streptococcus sanguis, Streptococcus salivarius, Streptococcus mutans, Aggregatibacter actinomycetemcomitans, Streptococcus mutans, Escherichia coli, Staphylococcus aureus, Staphylococcus epidermidis, S. saprophyticus, Streptococcus sanguinis, Lactobacillus vulgaris* and *Candida albicans*.^21^ Albabtain et al^1^ showed that benzyl isothiocyanate (a chemical substance released from miswak upon chewing) and essential oils were cytotoxic towards gingival fibroblasts; if using the miswak stick more than once, it is recommended to cut the tip before each use and use it immediately.^1^

The study results were in accordance with the Gazi et al^13^ and Al-Otaibi et al,^6^ who found that miswak mouthwash can statistically significantly decrease gingivitis and bleeding on probing compared with the conventional toothbrush. Besides, the results agreed with the studies by Al-Bayaty et al,^2^ Prasad et al,^23^ and Gupta et al,^14^ who report that plaque was reduced with the use of miswak mouthwash. Furthermore, miswak mouthwashes showed no statistically significant difference from regular mechanical plaque measures such as brushing. Therefore, it is recommended to support mechanical plaque control with mouthwash usage.^4^ On the other hand, the use of miswak may be related to gingival recession.^15^

The miswak mouthwash tested in this study was a mixture of the active component (miswak) dissolved in water, not in an alcoholic solvent in order to avoid any possible irritation to the gingiva. However, flavouring, colouring and sweetening agents are needed to make the mouthwash more palatable.^12^ Miswak mouthwash is a more advisable form than the sticks or toothpaste, due to its rapid therapeutic effects and targeted application on the oral cavity mucous membranes and gingiva.^3^

Miswak mouthwash can be considered as an affordable choice in cases such as uncooperative children, low socioeconomic status, disability, lack of manual skill and motivation, and special-needs individuals who are at risk of accidentally swallowing fluoridated toothpaste or chemical mouthwash formulations.

## Conclusion

The efficacy of miswak mouthwash is lower than that of CHX mouthwash. However, it can be an option for enhancing the oral health of people of different ages, health conditions and socioeconomic status, especially for long-term use because of its safety, efficacy, cost-effectiveness, ease of implementation, and availability.
